# Patients with head and neck cancer treated with radiotherapy: Their experiences after 6 months of prophylactic tooth extractions and temporary removable dentures

**DOI:** 10.1002/cre2.418

**Published:** 2021-03-23

**Authors:** Carl‐Otto Brahm, Carina Borg, Dan Malm, Bengt Fridlund, Freddi Lewin, Ahmed Zemar, Peter Nilsson, Apostolos Papias, Maria Henricson

**Affiliations:** ^1^ Department of Specialist Dental Care Public Dental Service Skövde Sweden; ^2^ Department of Natural Science and Biomedicine School of Health and Welfare, Jönköping University Jönköping Sweden; ^3^ Department of Oral and Maxillofacial Surgery Institute for Postgraduate Dental Education Jönköping Sweden; ^4^ Department of Nursing Sciences School of Health and Welfare, Jönköping University Jönköping Sweden; ^5^ Department of Heart Disease Haukeland University Hospital Bergen Norway; ^6^ Department of Oncology Ryhov County Hospital Jönköping Sweden; ^7^ Department of Orofacial Medicine Public Dental Service Linköping Sweden; ^8^ Department of Prosthodontics, Faculty of Odontology Malmö University Malmö Sweden

**Keywords:** head and neck cancer, prophylactic tooth extractions, radiotherapy, temporary removable dentures

## Abstract

**Objectives:**

The impact of dental occlusion on the experiences of head and neck cancer patients and their oral, social and psychological functioning has been sparsely investigated. There is a lack of knowledge regarding the experience of tooth loss and dentures among patients treated for head and neck cancer. The aim of this study was to describe the experiences of head and neck cancer patients of prophylactic tooth extractions and temporary removable dentures, 6 months after radiotherapy treatment.

**Material and methods:**

An individual interview with 25 patients 6 months after radiotherapy was subjected to a qualitative content analysis.

**Results:**

Two categories, *Impaired oral function* and *Belief in the future*, and seven subcategories described the patients' experiences of temporary removable dentures during the first 6 months after prophylactic tooth extractions. The temporary removable dentures affected the patients' ability to chew, swallow and speak, caused pain, and were experienced as an enemy. Despite that, the patients were hopeful and had a wish for recovery, which gave them the energy to live.

**Conclusion:**

Prophylactic tooth extractions and temporary removable dentures 6 months after radiotherapy treatment affect head and neck cancer patients' recovery and everyday life. However, they have the will to take on these challenges, pertaining not only to themselves, but also to relatives and health professionals. At the individual level, the patient needs individualized professional support to get through the arduous procedure, from the acute situation until the end of the rehabilitation phase.

## 1 BACKGROUND

The global annual incidence of head and neck cancer (HNC) was 650,000 cases in 2018 (Bray et al., 2018; Ferlay et al., 2019). In Sweden, the number was 1500 cases, accounting for 2.3% of all malignancies. The majority of cases were male, with a few exceptions; for oral cancer and cancer of the salivary glands, the gender distribution was more even (SweHNCR, 2019). In the majority of these cancers, the oncological treatment, including radiotherapy, involves the oral cavity. Therefore, patients are referred to a dentist for a pre‐treatment assessment, including treatment of dental pathologies, within eleven days of the multidisciplinary conference. This assessment is performed in order to reduce the risk of the severe side effect of osteoradionecrosis of the jaws (ORNJ), due to hypovascularity and reduced healing (Kunskapsbanken, 2018; Muraki et al., 2019; Willaert et al., 2019). Radical dental treatment, such as prophylactic tooth extractions (PTE) (Lockhart & Clark, 1994; Turner, Mupparapu, & Akintoye, 2013), may impair both the patient's appearance and oral functions, such as chewing and swallowing (Clough, Burke, Daly, & Scambler, 2018; Parahoo, Semple, Killough, & McCaughan, 2019). The Swedish National Board of Health and Welfare provisions (Hedegran, 2010) regarding HNC patients allow the replacement of extracted teeth by temporary removable dentures (TRD) at health care fees.

To date, the impact of dental occlusion on the experiences of HNC patients and their oral, social and psychological functioning have been sparsely investigated. There is a lack of knowledge regarding the experience of tooth loss among patients treated for HNC (Clough et al., 2018; Parahoo et al., 2019). So far, only two studies have problematized the experiences of tooth loss from a qualitative methodological perspective (Clough et al., 2018; Parahoo et al., 2019), and both found a negative impact of tooth loss on the patients' oral, social and psychological functions. The patients also experienced the time between being told that teeth had to be removed and the actual extractions to be very short, and that the information given or the opportunities to ask about alternatives were insufficient. The dentures were difficult to manage and caused problems of their own in terms of eating, speaking and appearance and clear pathways regarding post‐treatment prosthodontic rehabilitation were proposed (Parahoo et al., 2019). TRD often have inadequate function and cause discomfort (Siadat, Alikhasi, & Beyabanaki, 2017). Accordingly, there are no research to find about the impact of TRD after PTE among HNC patients and there is a knowledge gap regarding the experiences of HNC patients of temporary prostheses after PTE.

## 2 OBJECTIVES

The aim was to describe the experiences of HNC patients of PTE and TRD, 6 months after radiotherapy treatment.

## 3 MATERIAL AND METHODS

### 3.1 Design and context

The present study is a part of a prospective, multicenter quasi‐experimental study including five clinics which was carried out at the Departments of Special Care Dentistry and Oral medicine in Public Dental Care, in three medium‐sized and a larger region in the south of Sweden. The intervention was evaluated with regard to the impact of fixed dentures in HNC patients, in order to restore oral functions after PTE. In these patients, chewing function was initially restored with TRDs and finally with permanent fixed dental prostheses. The restoration process was evaluated through interviews at 6 and 12 months after the end of radiotherapy. The interviews at 6 months are in focus in this study, and were subjected to a qualitative content analysis, according to the study's aim (Hsieh & Shannon, 2005). Data from the evaluation of the permanent prosthodontic treatment 12 months after the end of radiotherapy will be published elsewhere. The patients were registered in the HNC fast‐track program (Kunskapsbanken, 2018), initiated by an otolaryngologist, and the investigation phase prior to the multidisciplinary conference was completed within two weeks. A conference decision on the diagnosis, TNM classification (T primary tumor site, N regional lymph node involvement, M presence of metastatic spread) (Brierley, Gospodarowicz, & Wittekind, 2016) and HNC treatment plan was the starting point of the dental treatment. Within another eleven days, teeth with a poor prognosis had to be extracted and the soft tissue healed prior to the oncological treatment planning. The patients were then referred to a prosthodontic specialist clinic. The prosthodontic treatment with TRD was initiated prior to or approximately 1 month after completed radiotherapy. Patients were informed about the consequences of tooth extractions, side effects of radiotherapy on oral health, and the consequences of TRD.

### 3.2 Participants

Patients diagnosed with a malignant tumor of the head and neck area and scheduled for radiotherapy, with or without surgery or chemotherapy, were included. No jaw resection patients were included in the study. The oncological prognosis at inclusion was initially good, and patients had one or more infected teeth extracted prophylactically to reduce the risk of ORNJ. Patients with communication difficulties due to mental or physical disability, other concomitant diseases with a poor prognosis, poor oncological prognosis initially, a predominant risk of tumor recurrence after completed radiotherapy, and biological or technical obstacles that made rehabilitation with fixed prostheses impossible, were excluded. In total, 43 intervention patients were included in the descriptive study, but six died, nine were excluded and three declined interviews for social reasons, resulting in a total of 25 patients (Table 1). Background factors, such as gender, age, tumor diagnosis, dry mouth, maximum jaw opening, difficulty swallowing, weight, positions, and the number of extracted teeth, were recorded. Three of the patients used dentures at baseline (patients no 5, 7 and 9).

**TABLE 1:** Demographic patient data. Gender was stated as female (F) or male (M), and age in years

Patient	Gender	Age	Diagnosis	Occupation	Prophylactic tooth extractions (*n*)
1	M	63	Palatal cancer, T3N2M0	Chimney sweep	7
2	F	59	Tonsil cancer, T1N2aM0	Preschool teacher	1
3	M	69	Base of tongue cancer, T1N0M0	Retired	5
4	M	67	Lymphoma of the neck	Mechanic	1
5	M	76	Cancer with unknown primary, TxN2M0	Industrial worker	2
6	F	74	Lip cancer, T2N1M0	Receptionist	3
7	M	94	Parotid cancer, T2N0M1	Haulier	2
8	M	80	Cancer with unknown primary, TxN2M0	Toolmaker	6
9	M	81	Parotid Muco‐epidermoid cancer, T2N0M0	Property manager	14
10	F	56	Tonsil cancer, T2N1M0	Health and social care assistant	8
11	M	43	Parotid Muco‐epidermoid cancer, T1N0M0	Teacher	3
12	M	63	Nasal cancer, T4NxM0	Florist	6
13	M	67	Buccal cancer, T1N0M0	Production manager	2
14	M	76	Tonsil cancer, T1N0M0	Journalist	3
15	F	69	Cancer with unknown primary, TxN1M0	Health care assistant	1
16	F	66	Tonsil cancer, T2N2bM0	Health care assistant	2
17	M	71	Cancer with unknown primary, TxN2bM0	Municipal worker	1
18	M	60	Tonsil cancer, T2N2M0	Truck driver	3
19	F	75	Tonsil cancer, T1N1M0	Bank employee	4
20	M	63	Cancer with unknown primary, TxN2bM0	Mechanic	12
21	F	67	Tonsil cancer, T3N2bM0	Nurse	4
22	M	83	Ear Basal Cell Carcinoma, regional lymph node metastasis	Owner of dry cleaning shop	2
23	F	67	Lacrimal Gland Cancer	Student counselor	2
24	M	87	Buccal Cancer, T2N0M0	Farmer	3
25	M	52	Tonsil cancer, T2N2bM0	IT technician	3

*Note*: Diagnosis according to the TNM classification, 8th ed (Brierley et al., 2016).

### 3.3 Data collection

The semi‐structured interviews (Polit & Beck, 2016) with the intervention patients were performed by a dental assistant (C.B.), an employee at one of the specialist dental clinics, who had good skills in performing qualitative interviews, was well acquainted with HNC patients, and used to talking to patients in difficult situations. This was important to ensure an understanding of the patients' experiences of temporary removable dentures, and to be able to ask relevant follow‐up questions. The interview guide was developed based on previous research and the aim of the study and containing six items: the treatment, oral function, social life, emotions, thoughts about the future, quality of life. Patients were contacted by C.B. after 3 months of using the dentures, approximately 6 months after the end of radiotherapy, to make an appointment for an interview. The interviews were coordinated with routine check‐ups at the clinic between March 2013 and October 2019 and were conducted undisturbed in a private room. One telephone interview was conducted as the patient had moved to another region. The interviews lasted up to 60 minutes, were recorded on Minidisc and transcribed verbatim, including non‐verbal expressions such as crying, laughter and breaks (Polit & Beck, 2016). The interviews began with an open question, “*Please tell me about your experiences from the first visit you had here until today*”. Patients were encouraged to talk about their experiences of temporary dentures and follow‐up questions were asked, if needed, to achieve a deeper understanding. Data collection was preceded by three test interviews for practice, which resulted in an adjustment of the open question. The three test interviews were not used due to lack of content. The data collection continued until data saturation was achieved, involving a total of 25 patients, as no new information based on the study's aim emerged in the interviews.

### 3.4 Data analysis

A qualitative manifest content analysis according to Hsieh and Shannon (2005) was made to find variations in the content of the interviews. All interviews were listened to and checked against the printouts to ensure credibility. Furthermore, each transcript from the interviews was read through several times before C.B., who made the analysis in cooperation with M.H., highlighted the text that described the patients' experiences of PTE and TRDs and wrote keywords or a phrase in the margin of the text that captured the experience in the patient's own words. In total, 484 keywords were selected, which were then condensed and given a code that characterized the core of what the keyword described. The codes were laid out next to each other to identify similarities and differences and were then condensed into subcategories (Table 2). In this way, seven subcategories were created that were tested against the aim, the keywords and the codes that made up the subcategory. Finally, the whole research group reached consensus about two categories. The research group's pre‐understanding of the context was considered and reflected according to a multidisciplinary background within health care in general and oral health care in particular.

**TABLE 2:** Example of the analysis process

Keywords	Codes	Subcategories	Categories
*“It just feels like YUK! I don't like having it in my mouth! I poke around with my tongue, and then I go shopping and on my way home, as soon as the coast is clear, it goes into my pocket.” (Patient 6)*	TRD friend or enemy	TRD as the enemy	Impaired oral function
*“We haven't kept anything secret and then you feel some kind of response to being open, friends dare ask questions and they have been there for us.” (Patient 21)*	Socially	The energy to live	Believe in the future

### 3.5 Ethical considerations

The ethical process was conducted in accordance with the World Medical Association's Declaration of Helsinki (Human & Fluss, 2008), and approved by the ethical review board in Linköping (Reg. no: 2012/200:31). Informed consent was achieved by all participants.

## 4 RESULTS

**FIGURE 1:**
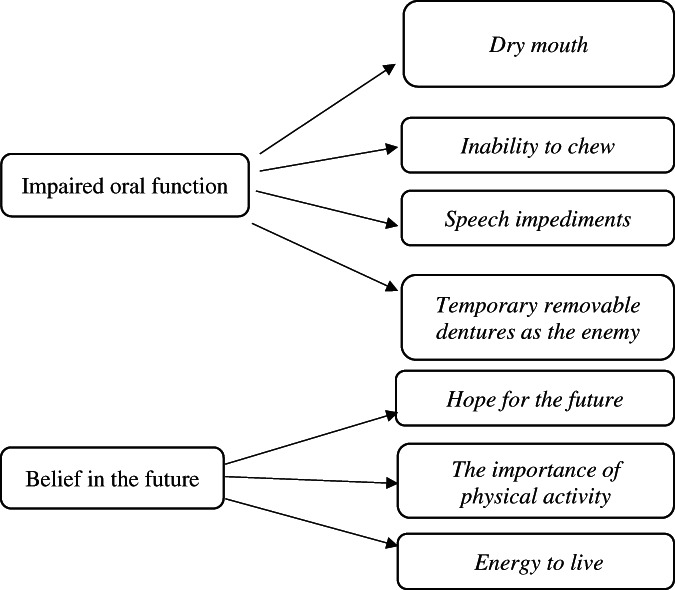
Overview of categories and subcategories describing the patients' experiences of prophylactic tooth extractions and temporary removable dentures 6 months after radiotherapy treatment

Two categories and seven subcategories emerged that described the patients' experiences of TRD during the first 6 months after PTE. The categories were *Impaired oral function* and *Belief in the future* and the internal relationships with the subcategories are shown in Figure 1. The PTE and TRD affected the patients' ability to chew, swallow and speak, caused pain and were experienced as an enemy. Despite that, the patients were hopeful and had a wish for recovery, which gave them the energy to live.

The focus on the complaints from the oral cavity of radiotherapy was most pronounced during the first 2 months. The symptoms manifested themselves as oral mucositis (inflammation and ulcerations of the oral mucosa). Dry mouth, on the other hand, became later notable as a persistent problem for the patients.

### 4.1 Impaired oral function

This category described patient experiences, such as dry mouth, increased need for oral self‐care, difficulties with chewing and swallowing and pain, after tooth extraction(s), oncological treatment and TRD use.

#### 4.1.1 Dry mouth

Dry mouth was a consequence of radiotherapy that had a negative effect on the patients' ability to eat. Impaired sense of taste and difficulty swallowing due to dryness of the mouth lead to weight loss, among other things, even though the patients forced themselves to eat at every meal. The TRD in the mandible was felt with the tongue, making it taste of plastic, and the dentures were perceived as moving and shifting position when biting onto something hard or tough. Some patients felt as if they had dough in the mouth when they ate a sandwich and adding liquid was necessary for swallowing. The experiences of self‐care of oral health and of maintaining good oral care after meals was described as almost a full‐time job.

“*Brushing the teeth three times a day and then using fluoride once a day and then there were other things as well. Then I had to take something for the fungus four times a day… it was driving me up the wall…*” (Patient 3)

Dry mouth as a side effect of radiotherapy also caused problems with oral fungal infections, which also impaired the patients' appetite and desire to eat. The meal was just a process to ingest food, and the patient would have preferred to stop eating. A feeling of being constantly cold with swelling and thickening of the throat was described. Sleep was also affected, which was described very clearly by a patient.

“My mouth feels so dry, so some nights I wake up 3‐4 times and then I'm extremely tired when I have to get up. I also have nights when I only wake up once and sleep really well, and then I feel a lot better and have more patience and feel mentally better too.” (Patient 2)

There were both patients who did and who did not experience dry mouth during the time using a TRD.

#### 4.1.2 Inability to chew

Chewing ability was impaired by PTE and TRDs. The patients felt that food got stuck everywhere in the mouth and everything was turned into a mess. Furthermore, they described difficulty chewing the food, which made them swallow too large pieces that upset the stomach. There were also problems with chewing small pieces of food, as the impaired motor skills and perception made the patients feel that they did not “get hold of the food”, thus not getting the reflex to swallow. The patients experienced that the TRD had to be removed at mealtimes as it was impossible to eat with it, and the wait for a fixed prosthesis felt long.

“*I thought we would begin putting in a fixed prosthesis soon. But I feel that time is dragging on a bit; I can't wear them when I eat but have to remove them*.” (Patient 7).

Patients felt ashamed when removing the dentures at meals with food they could not chew. Someone held a napkin in front of the mouth so as not to embarrass him/herself and others at the dining table when removing the TRD.

“*It would be best if I could eat since there are so many social aspects to that. I don't want to visit anyone and I'd rather no one came to see me either, when there is food involved and that. It does feel a bit weird; they can come to my place for a coffee, that's fine, but when it comes to food and I can't eat it doesn't feel great.” (Patient 16)*

If the patients were invited to somebody's home or to a restaurant, vegetables had to be properly cooked and soft, otherwise they could not be broken up by chewing and swallowed. Eating small pieces of food was easier, but then it took longer to finish the meal, which was difficult when being together with other people.

#### 4.1.3 Speech impediments

The PTE and TRD imposed speech impediments on the patients, who experienced blurring or lisping and the TRD coming loose during conversations, which was embarrassing and made the patients greatly aware of the dentures. Also, the TRD changed the ability to form sounds and to pronounce vowels in the correct way, which made it difficult for patients in their professional roles. One patient described how teaching her students the correct pronunciation was affected:

“*It's mainly that I feel that it's in the way in the palate so it's more difficult, and I'm working a lot with something called Fonomix with the children in nursery school. It's about speech sounds, teaching the children about the sounds, that it's not the letter K but KE and some of the sounds are more difficult to make when I wear the dentures. I didn't feel like that before.” (Patient 2)*

The radiotherapy also affected the vocal cords so that the voice changed, became hoarse and raspy.

#### 4.1.4 The TRD as the enemy

Patients experienced a great deal of frustration with a TRD, as it chafed and loosened, and food got stuck underneath and caused pain while chewing. It was therefore a relief to remove it as soon as they came home. There was a major difference between having a fixed dental prosthesis and a TRD, as the latter felt plastic and loose all the time and the clasps irritated the gums. For one patient, the frustration was great:

“*It just feels like YUK! I don't like having it in my mouth! I poke around with my tongue, and then I go shopping and on my way home, as soon as the coast is clear, it goes into my pocket*.” (Patient 6).

Patients expressed sadness about having lost several teeth and found it difficult to use a TRD. They did not want to be dependent on eating soups and soft foods because of the TRD. The dentures felt slimy, tasted of plastic and constantly produced an unpleasant feeling in the mouth. The tongue was continuously seeking out the space between the teeth and the TRD. Also, the TRD felt as if it was moving and shifting positions when biting onto something hard or tough.

### 4.2 Belief in the future

Although the patients experienced a hard time with the PTE and TRD, there was hope for general improvement in the future, which gave them the energy to perform physical activities and to go on living.

#### 4.2.1 Hope for the future

The patients expressed greater belief in the future and stronger feelings of joy and hope when told that everything was fine at the oncological post‐treatment check‐up: “*It's the best [news] a person can get*.” *(Patient 11)* At first, the patients felt that it was a tough diagnosis but that it was important to know that things were going in the right direction. Getting help, support and a good response from doctors, dentists and their families made the patients feel that they were moving on and would continue to do so in the future. The dietician, who called every week to check the patient's weight, was also an important person.

It was important to be able to feel hopeful about the future, of a life together with wife/husband and of watching the grandchildren grow up. The patients hoped the disease would be gone after the oncological treatment, but it was difficult to know if that would be the case. Life shifted between hope and despair.

“… *because it was only the tongue in the beginning and then he said, he thought it was ok after the tongue, but that you never know where it's going. And that is something that I have thought about on and off, that this might spread.” (Patient 3)*

When patients were informed about their cancer, there was only one thing on their mind: to fix it and to follow the doctor's and dentist's advice to cope with the disease. On top of that, being told to pull out teeth made the patients think, “*OK, so these are the rules of the game, I have to do this to fix my tumor*.” *(Patient 9)* The patients reflected over why they had been lucky enough to survive, at the same time as they worried about the cancer coming back.

#### 4.2.2 The importance of physical activity

It was important to be able to move around, take walks, play golf with friends and have an active life, like before [the cancer], since these activities were not affected by wearing a TRD.

“*Before the cancer, when I was at home, I was never inactive. There was the garden to look after and all that, and I would take 3km walks, but then this hit me, and it has reduced my energy considerably.” (Patient 7)*

Those patients who were able to find peace of mind and feel better from being close to nature, from fishing, hunting and walking in the woods, experienced some pleasure during the disease period. However, the lack of saliva production was described as a problem in connection with physical activity and exertion, as the patients became breathless and experienced dryness of the mouth when breathing. A small spray can with water relieved the discomfort, and one patient reported that he always brought it when walking his dogs; if not, he had to turn back home to fetch it because he could not stand his dry and sticky mouth.

#### 4.2.3 The energy to live

The urge to recover gave the patients the energy to live. Some patients experienced post‐treatment fatigue that made it impossible to work full time. Other patients experienced mental stress about going back to work, as they felt that they could not contribute or didn't do anything useful, just “made an appearance”. In order to have the energy to live, it was important for the patients to share their feelings and not be afraid of telling their family and friends that they were unhappy.

“*We haven't kept anything secret and then you feel some kind of response to being open, friends dare ask questions and they have been there for us*.” (Patient 21).

For some patients, supportive family members and friends came along to the radiotherapy treatment sessions and medical appointments, even though they felt sad and worried about the patient's health. The patients expressed a desire that the health service would also look after the relatives in this situation, since all the focus is on the patient with cancer.

## 5 DISCUSSION

The aim was to describe the experiences of HNC patients of PTE and TRD, 6 months after radiotherapy treatment. The main findings show that HNC patients have the will to live and to endure the side effects of radiotherapy and of the use of a TRD that affected their oral, social and psychological functions negatively. Despite the oncological diagnosis and treatment message, the HNC patients described that they had the will and courage to meet the challenges that the dental treatment would pose. In that situation, they not only had their own distress to deal with, but also had to cope with the emotional and social needs of their family and friends.

According to the Swedish HNC fast‐track program (Kunskapsbanken, 2018), patients were referred from the ENT clinic to the specialist dental care clinic for a dental examination on the same day as the multidisciplinary oncological conference took place. In the present study, patients described that during the investigation phase they accepted their situation and had confidence in the health service and the dental staff making the right assessments. This was a survival strategy that they only later understood and experienced the consequences of. None of the patients questioned the PTE prior to radiotherapy, unlike in the study by Parahoo et al. (2019), where the patients were surprised by the recommendation to remove some or all their teeth when receiving primary radiotherapy. Later, they were sad about having lost their teeth, in addition to the uncertainty about whether they were cured or not and the fear of the cancer recurring. This is in accordance with Clough et al. (2018) whose patients described grief about having lost their teeth prior to radiotherapy, and who felt that they had not been involved in the decision to extract the teeth. According to Parahoo et al. (2019), losing teeth that the patients perceived as being healthy, had profound negative effects on their lives in terms of eating, speaking and social interaction.

In accordance with a Danish‐Swedish study, a range of different impairments due to tooth loss and strategies to accommodate them, was also found for partially edentulous patients that had no recent cancer treatment (Øzhayat, Akerman, Lundegren, & Owall, 2016). The impairments were mostly experienced as problems manifesting themselves in social settings. The participants were afraid of being judged negatively and therefore felt limited in specific social situations, such as during job interviews and when dating. Øzhayat, Korduner, Bagewitz, and Owall (2020) described coping strategies for functional, aesthetic and social impairments, and coping strategies and modifications of the impairments affecting partially edentulous patients with no history of recent cancer treatment from two prosthodontic specialist clinics in Sweden.

Concern shown by care staff, good service and coordination during the treatment and the dental visits were extremely important for these patients and their relatives, as their life situation was as stressed as it gets in a life‐threatening illness. Dental health professionals who were part of the HNC multidisciplinary team contributed to a better life situation for the patients. Semple, Dunwoody, Kernohan, McCaughan, and Sullivan (2008) underlined that the dental staff meet the patient during an extremely sensitive period, that is, shortly after the cancer therapy, that multidisciplinary care is advantageous, and that there is a need for individualized care.

The side effects of radiotherapy varied but most patients had severe impairment of their oral functions during the first 2 months after completed oncological treatment. Similar results have been described in Hollander‐Mieritz et al. (2019), where the most frequently mentioned symptoms were oral pain, decreased appetite, dysphagia, dry mouth, fatigue and hoarseness. However, the side effects of radiotherapy in the oral cavity varied over time and so did the patients' experiences and focus on oral problems. Furthermore, during the oncological treatment, the HNC patients worried about the emotional and social needs of their family and friends that were not taken care of professionally, which, to our knowledge, has not been reported elsewhere.

During the rehabilitation phase, patients described it as essential to learn how to live with and accept a “new” oral cavity and appearance that would never be the same as before the cancer treatment. The same was true of prosthodontic rehabilitation, that is, learning to use and accept a TRD. The ability to readjust among HNC patients has been reported elsewhere (Semple et al., 2008). The extracted teeth were replaced with a TRD prior to or approximately 1 month after completed radiotherapy. Although it was difficult to use the TRD, the patients remained positive about having an aesthetic replacement in order to feel comfortable in social situations. Nevertheless, patients did not want to eat with family and friends, as they felt embarrassed about not being able to chew and swallow properly, about behaving inappropriately, and about taking too long to eat and having to leave the table to clean the dentures. Chewing difficulties have been described frequently for HNC patients (Einarsson, Laurell, & Tiblom Ehrsson, 2019; Ganzer, Touger‐Decker, Byham‐Gray, Murphy, & Epstein, 2015; Jager‐Wittenaar et al., 2011; Ottosson, Laurell, & Olsson, 2013; Parahoo et al., 2019). Problems with speaking were also described by patients, in agreement with previous studies (Fromm, Gotfredsen, Wessel, & Ozhayat, 2019; Parahoo et al., 2019). Moreover, patients experienced challenges with the dentures, such as poor fit, feeling of discomfort in the gums and the dentures falling out when eating, which caused problems with eating, speaking and appearance. In accordance with Parahoo et al. (2019), physiological, functional, emotional and social functions were affected by the dentures, with the consequence that patients need support in different ways to cope with health and life.

### 5.1 Methodological considerations

To improve trustworthiness, the components *credibility*, *objectivity*, *dependability*, and *transferability*, were used in the checklist by Elo et al. (2014). Credibility can be considered both a weakness and a strength, since the person who recruited patients to the study, the second author C.B., employed by one of the specialist dental clinics, knew the patients from the dental examination but did not take part in the dental treatment during the study. In order to establish *objectivity*, C.B., who conducted the interviews and made the analysis, dealt with her pre‐understanding by peer debriefing and communication with the last author (M.H.), who had limited experience of the topic but extensive methodological experience. The experience of the topic was based on C.B.'s professional role as a dental assistant. *Objectivity* was achieved by audio recording the interviews and transcribing them verbatim, taking a critical stance during the analysis and presenting quotations from the participants. The fact that three test interviews were performed for practice, and that one researcher conducted the interviews strengthened the *objectivity*. The participants represented different ages, professions, and had extracted varying numbers of teeth, enhancing the *dependability* of the content analysis, which aims to include as many variations as possible. A limitation was the lack of a specific diagnosis and tumor location, which may have affected the trustworthiness of the study. The context, selection and characteristics of the participants, data collection and analysis process were described in order to facilitate comparison with other studies, which strengthened the *transferability*.

## 6 CONCLUSION AND CLINICAL IMPLICATIONS

PTE and TRD 6 months after radiotherapy treatment affect HNC patients' recovery and everyday life. However, they have the will to take on these challenges, pertaining not only to themselves, but also to relatives and health professionals. At the individual level, the patient needs individualized professional support to get through the arduous procedure, from the acute situation until the end of the rehabilitation phase. The same actions are also very relevant at the dyadic level, to deal with affected partners and relatives. At the organizational level, it is obviously advantageous to use multi‐professional health competence during the entire procedure, as specific skills contribute to synergies in a professional support program. The general impact on health and life of the PTE and the TRD is further evaluated in the quasi‐experimental follow‐ups of the radiotherapy treatment at 6 and 12 months, bringing greater clarity about how such professional support models and tools should be designed for the clinical setting.

A need for oral and written information to the patients prior to treatment is apparent regarding the consequences of tooth loss and negative experiences of TRD. Fixed restorations seemed to be preferred by HNC patients treated with radiotherapy, but further studies are needed.

## CONFLICT OF INTEREST

The authors have no conflict of interest to declare.

## Data Availability

Data available on request due to privacy/ethical restrictions.
